# Supplementary material for: MetaRibo-Seq measures translation in microbiomes
Source: Nat Commun. 2020 Jun 29;11:3268. doi: 10.1038/s41467-020-17081-z (PMC7324362; doi:10.1038/s41467-020-17081-z)
Supplement: Supplementary file 10 — Supplementary Data 7 [file 41467_2020_17081_MOESM10_ESM.zip › File2/Confidence_High_Taxonomy/320400_out.krona.html]

Javascript must be enabled to view this page.

members
magnitude
magnitudeUnassigned
count
unassigned
taxon
rank

320400\_out

8

superkingdom
2
8

8
976
phylum

class
8
200643

order
171549
8

171552
8
family

genus
838
8


SRS077849\_contig\_number\_11621
species
59823
1

species
7
165179

SRS012969\_contig\_number\_46SRS012989\_contig\_number\_9449SRS023914\_contig\_number\_26523SRS043841\_contig\_number\_5643SRS077194\_contig\_number\_405SRS1041134\_contig\_number\_10912SRS146764\_contig\_number\_31953
